# Foliar Nutrition Influences Yield, Nut Quality and Kernel Composition in Hazelnut cv Mortarella

**DOI:** 10.3390/plants12112219

**Published:** 2023-06-04

**Authors:** Antonio Pannico, Giuseppe Carlo Modarelli, Silvia Rita Stazi, Matteo Giaccone, Raffaele Romano, Youssef Rouphael, Chiara Cirillo

**Affiliations:** 1 Department of Agricultural Sciences, University of Naples Federico II, 80055 Portici, Italy; antonio.pannico@unina.it (A.P.); giuseppecarlo.modarelli@unina.it (G.C.M.); raffaele.romano@unina.it (R.R.); youssef.rouphael@unina.it (Y.R.); 2 Department of Chemical, Pharmaceutical and Agricultural Sciences, University of Ferrara, 44121 Ferrara, Italy; silviarita.stazi@unife.it; 3 Institute for Mediterranean Agricultural and Forest Systems, ISAFOM, National Research Council of Italy (CNR), Piazzale Enrico Fermi 1, 80055 Portici, Italy; matteo.giaccone@cnr.it

**Keywords:** nitrogen, microelements, boron, fatty acids, polyphenols

## Abstract

In hazelnut, foliar nutrition is utilized globally to integrate microelement deficiencies and optimize their assimilation and effects on yield performances. Nevertheless, nut quality and kernel composition can be positively affected by foliar nutrition. Recently, several studies pointed out the need for increasing the sustainability of orchard nutrition by proposing the management of not only micronutrients, but also main components, such as nitrogen, through foliar spraying. In our study, different foliar fertilizers were used to understand the effectiveness of supporting hazelnut productivity and nut and kernel quality. Water was used as a control. Foliar fertilizations affected tree annual vegetative growth, improved kernel weight and decreased the incidence of blanks compared to the control. Differences in fat, protein, and carbohydrate concentration were also found among treatments, with increased fat concentrations and total polyphenols content in fertilized treatments. Foliar fertilization improved the oil composition of the kernels, though fatty acid composition responded differently to nutrients spray. Oleic acid concentration was promoted, while palmitic acid concentration was reduced in fertilized plants compared to control trees. Furthermore, CD and B trees were characterized by an increase in the ratio of unsaturated/saturated fatty acids compared to untreated trees. Finally, foliar spraying improved lipid stability compared to the control due to higher total polyphenol concentration.

## 1. Introduction

In the last 20 years, hazelnut cultivation expanded to various areas of the world [1]. This increase was mainly driven by the confectionery industry, which also opened up to new markets. Nevertheless, the expansion to new areas and the increased pressure of climate change highlighted the need to improve cultivation techniques to increase the efficiency of orchards. Among the various agronomic practices, fertilization certainly plays a key role. A correct supply of nutrients improves plants’ vegetative and productive efficiency. Numerous studies on various plant species highlighted the importance of “micronutrients”, such as iron, boron and zinc, and “macronutrients” in optimizing yield and nut quality. The ordinary fertilization techniques involve the incorporation of fertilizers into the soil. However, roots’ nutrient absorption abilities depend on several environmental factors, such as water availability in the soil [2], particular soil reaction [3] and soil aeration conditions [4]. In addition, in mature orchards, adult trees increase the depth of newly produced roots, with those with better absorption capacity, thus, resulting in less efficient nutrition. In these cases, foliar fertilization becomes an important tool to support granular soil fertilization [5].

Due to the spread of hazelnut cultivation to various areas of the world, it is necessary to reassess orchard management practices, including the nutritional requirements and nutrient application methods [6]. In addition, the hazelnut mineral nutritional requirements were defined for the most well-known and used varieties in the traditional productive areas [7]; however, data are missing for new cultivation contexts, thus requiring deeper insight.

The mineral nutrition of the hazelnut tree has important effects on nut yield and quality [8]. Like for other tree crops, nitrogen is the macronutrient for which greater accuracy of management is required in the quantities administered, as it is particularly mobile and influences the plant’s vegetative activity [9]. Nitrogen also plays an important role in kernels’ development, reaching contents higher than 5% of the dry mass during its filling phase, thus suggesting the importance of nutrient availability during this physiological phase [9].

Among the microelements, boron is also crucial for growth and reproductive development in nut plants, such as pecan [10], macadamia [11] and almond tree [12]. In hazelnut, boron is considered one of the essential nutrients for an excellent fruit set and for improving the nut quality [13,14]. Studies conducted in Oregon show that it increases fruit set and improves the quality of hazelnuts [15,16,17], while in Mediterranean cultivation conditions, boron is observed to have effects ranging from null to significant [13,18,19] on the reduction in blank fruits [20]. Further studies showed that foliar sprayings of boron with macro-nutrients, such as nitrogen, and other micronutrients, such as zinc and iron, improve the fruit set, yield and quality of hazelnuts [21,22,23,24,25,26], with the best results obtained using boron concentrations between 300 and 600 ppm [17,20,23].

On the other hand, in the climatic changes scenario, the application of mineral fertilization at the foliar level is particularly useful in conditions where the absorption of nutrients from the soil is limited, representing an additional way to supply nutrients during the critical growth phases [6].

The growing interest in foliar nutrition applied to hazelnut orchards revived some experimental activities to optimize intervention protocols, while also taking into account the varietal characteristics of the plants, as shown by recent acquisitions in “Tonda Gentile” [27], “Tonda Romana” and “Nocchione” [28], “Barcelona” [29], and various cultivars of American origin [21]. This intervention strategy, which is also being tested for applications of TFN (total foliar nutrition) to hazelnut orchards [28], is supported by scientific evidence that certifies how the leaves can rapidly absorb nutrients, contributing to a significant attenuation of the drifts’ phenomena and environmental pollution. Targeted foliar fertilization can also be carried out in a mixture with other substances, such as pesticides, osmolytes and biostimulants, to promote the resilience of the hazelnut orchard [30,31].

Based on these premises, this work aims to evaluate, for the first time, a complex foliar fertilizer, based on urea, organic nitrogen, boric acid, zinc EDTA, iron EDTA and a commercial product, known as Coryl Dry Veg and from now on referred to as CD, before comparing them to a foliar fertilizer based only on boric acid (B), which is usually adopted in soils where boron is scarcely available.

Therefore, a three-year trial was carried out to evaluate yield and some chemical quality parameters of the hazelnut (*Corylus avellana* L.) cultivar Mortarella. This cultivar is the highest quality not-rounded kernel cultivar grown in the Campania region, Italy, which is the second most important hazelnut-producing country in the world [1].

## 2. Results

### 2.1. Yield and Biometrical Characteristics of Nuts and Kernels

Foliar treatments affected yield components in 2013, since both the yield expressed in kilograms and the number of fruits per tree were higher in the CD-treated trees than B fertilized plants and C plants at harvest time (Table 1). In particular, the highest yield was harvested in 2013 × CD, and the lowest yield was harvested in 2011 × B. Similarly, the highest number of nuts per plant was recorded in 2013 × CD, and the lowest number was recorded in 2012 × C (Table 1). The percentage of blank fruits was lower in fertilized trees compared to the control in the years 2012 and 2013, while in the first year of the experiment, only the CD plants had the lowest incidence of blanks at harvest. The overall interaction Y × F indicated that the highest percentage of blank nuts was always recorded in the control plant, while the lowest percentages were observed in 2011 × CD and 2012 × CD plants, with B plants giving intermediate values in all three years of trial (Table 1).

The biometric characteristics of fruits and kernels at harvest showed significant differences between treatments in the three years of the experiment. The nut weight was mostly influenced by the year and not by the treatments, though the interaction Y × F indicated that only the C and B plants in 2011 produced the nuts with the lowest weight. Finally, the dry weight of kernels was affected by both the main factors. Indeed, in 2011, the lowest kernel dry weight was observed, and CD kernels showed a significantly higher dry weight than the control, while B treatment gave intermediate values (Table 1).

### 2.2. Leaf Mineral Composition 

Leaf mineral composition indicated that NH_4_, K and Ca concentrations in the leaves were only affected by the year of trial, whereas Mg concentration was also affected by the treatment (Table 2), with CD showing the highest value and C showing the lowest value. 

In contrast, all of the P and micronutrient concentrations measured were significantly affected by the interaction Y × F. Indeed, leaf P concentration was the highest in 2011 × CD, whereas it reached the lowest values in 2013 × control plants (Table 2), with all the other combinations showing intermediate results.

The highest Zn concentration was found in 2012 × B, followed by 2012 × CD and 2013 × CD, whereas the lowest concentrations were detected in all of the control treatments. (Table 2). The Fe concentration decreased from the first to the last year of the trial, and the overall effect of the treatments indicated an increased concentration in the leaves of the CD treated trees. However, the significant effect of the interaction Y × F highlighted that 2011 × B leaves had the highest content, whereas the lowest content was found in 2013 × C and 2013 × CD (Table 2). The B concentration increased with the years and, as expected, was highest in B treatment, lowest in the control leaves and reached intermediate values in CD (Table 2). Indeed, the Y × F interaction showed that the highest B concentration was found in 2013 × B leaves, followed by 2012 × B and 2011 × B, the CD treatments and, finally, the control leaves (Table 2). The Mn concentration was affected both by the year, decreasing from the first to the last year, and by the treatment, being highest in CD leaves (Table 2). In particular, the highest Mn concentrations were found in 2011 × CD and 2011 × B, followed by 2012 × CD, whereas the lowest values were recorded in all of the treatments from 2013.

### 2.3. Kernel Composition and Total Polyphenols Content

All kernel main constituents, which were analyzed on the yield of the years 2012 and 2013, were found to be similar between years, though the ash content was slightly reduced in the second year. In contrast, they were affected by foliar treatments (Table 3). In particular, the fat content was increased by 4.3% in CD and by 7.2% in B treatments compared to control (Table 3). The protein content percentage was the highest in CD (+31.8 %), while no differences were found between B and the control. As a consequence, the control showed the highest percentage of carbohydrates (22.94%), followed by B (−21.4%) and CD (−36.4%). Ash content was not affected by the treatments. 

**Figure 1:**
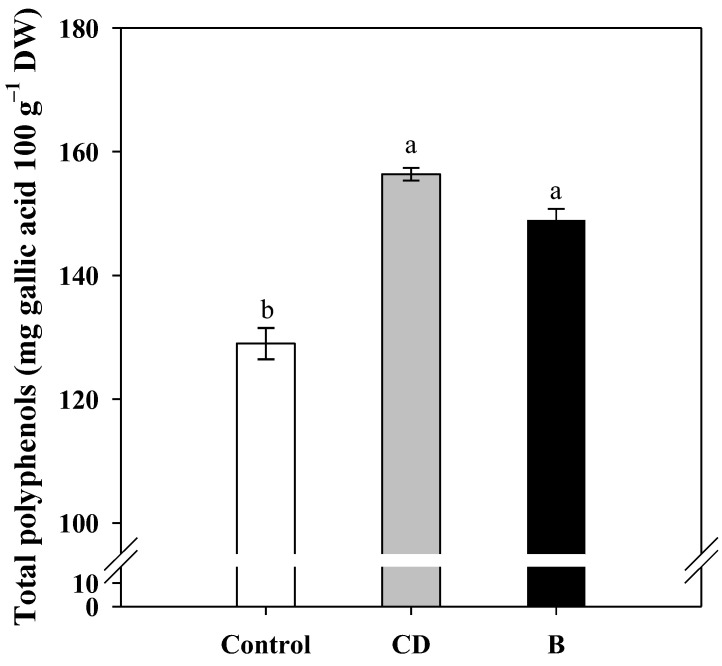
Total polyphenols content in oil extracted from hazelnut harvested in 2013 from foliar fertilized plants (CD and B) and control plants (C). Data are expressed as mean ± s.e., (n = 3). Different letters indicate significant differences according to Tukey’s HSD test (*p* = 0.05).

The total polyphenols content, which was determined based on the yield in 2013, was affected by foliar fertilization, with the kernels of CD and B treatments reaching higher values (153.4 and 147.7 mg 100 g^−1^, respectively) than the control (128.7 mg 100 g^−1^) (Figure 1).

### 2.4. Lipid Alteration

Foliar fertilization positively influenced the quality and the stability of the lipid fraction of hazelnuts (Table 4). In the last two years of the experiment, it was observed that the CD- and B-treated fruits had lower values for all parameters considered. Indeed, free acidity significantly decreased in all the treatments from the year 2013, with the lowest level found in the oil extracted by CD kernels (−57.8% compared to control of same year). In contrast, peroxide value was, on average, higher in 2013 than in 2012. However, in both cases, the oxidation grade of the oil was reduced by the foliar treatments compared to the control, with the highest level retrieved in 2013 × control treatment and the lowest level retrieved in 2012 × CD treatment (Table 4). Finally, the absorption coefficients (K_232_ and K_270_) showed a significant interaction Y × F, with the highest value found in 2013 × C (for both K_232_ and K_270_) and the lowest value found in both the 2012 × CD and 2013 × CD (K_232_) and 2012 × CD and 2012 × B (K_270_).

### 2.5. Oil Fatty Acid Composition 

From hazelnut oil analyses, we identified 13 different fatty acids. The year and the fertilization significantly affected most of the identified fatty acids; the only fatty acid not affected by either the year or the fertilization and by their interaction was C18:1n9. In addition, the year had no effect on the C16:0 and the C16:1 fatty acids, while the fertilization treatment had no effect on the C17:0, C17:1, C18:1n9 and C20:1 and C23:0. The interaction between the year and the fertilization significantly affected most of the fatty acids; the C16:0 did not vary within the fertilization treatments in the 2012, while its content increased in the 2013 × control and decreased in the 2013 × CD and 2013 × B. The C16:1 increased in the 2013 × control, while it decreased in the 2013 × B. The C18:0 was significantly higher in the 2012 × control and 2013 × CD than in the 2012 × B, and they decreased significantly in all the treatments in 2013, with the lowest values observed in the combination 2013 × CD. The C18:1n9 was lower in all the treatments in 2012 than in 2013, with the lowest values observed in the combination 2012 × control and 2012 × CD, while in 2013, they increased significantly, with the highest values observed in the combination 2013 × CD and 2013 × B. The C18:2n6 were similar between the fertilization treatments in 2012, while they increased in 2013 in the control and CD treatment and did not vary in the B treatment. The C18:3n3 in 2012 was lower in control plants than in CD and B plants, and, in 2013, they increased in control and CD plants while decreasing in B plants. The fatty acid C18:3n6 content was similar in 2012, while it decreased in 2013, with the lowest value registered in the combination 2013 × CD. The C20:1 did not vary between 2012 and 2013 in control and CD plants, while, compared to 2012, it increased significantly in B plants in 2013. The C20:3n6 was higher in 2012 than in 2013, and its content decreased significantly in control and CD plants, while it did not vary in B plants. Lastly, the C23:0 fatty acid was higher in 2012 than in 2013, and it decreased for all of the fertilization treatments in 2013. (Table 5). Relative content of fatty acids in the oil extracted from kernels of hazelnuts harvested in 2012 and 2013 from foliar fertilized (CD and B) and control plants was significantly affected by the Y × F interaction. In general, there was an increase in unsaturated fatty acids (UFA) in 2013 compared to 2012, with 2013 × CD reaching the highest content, followed by 2013 × B, while the lowest content was found in 2012 × C and 2012 × CD (Table 5).

Further differences were observed in the content of mono- (MUFA) and poly-unsaturated (PUFA) fatty acids (Table 5). In 2012, the B treated fruits had the highest percentage of MUFA (83.2% vs. 82.8% of control), while the CD treated fruits had greatest percentage of PUFA (7.4% vs. 7.3% of control). In 2013, the fertilized nuts obtained a higher percentage of MUFA (84%) compared to the control (83.5%), while CD treated nuts confirmed a greater percentage of PUFA (7.8% vs. 7.4% of control) (Table 5). Regarding the Omega 3 and 6 contents in the kernels, both levels were lower in 2012 than in 2013 and increased significantly in 2013 in control and CD plants, while in B plants, the Omega 3 content decreased in 2013 and the Omega 6 content did not vary (Table 5).

## 3. Discussion

In several areas where intensive cultivation of hazelnut is spreading, the use of foliar fertilizers recently attracted increased interest in the context of more sustainable orchard management practices [27,28]. Furthermore, the application of foliar sprays to integrate micronutrients such as boron became a common nutritional strategy in non-deficient hazelnut orchards according to the results of studies conducted in many areas of cultivation. However, a better understanding of the effects on the overall yield of integrated management of foliar nutrition based on the application of nitrogen and micronutrients, as well as the nut quality and composition of hazelnut trees grown in recently developed areas of cultivation, such as the wide plain of Campania Region of southern Italy, needed to be considered.

### 3.1. Effects of Foliar Fertilization on Leaf Mineral Composition and Yield Components 

In the present trial, foliar fertilizations applied to hazelnut trees of cv Mortarella, which were grown in volcanic-origin acid soils, induced some direct effects on leaves’ concentrations of macro- and/or micro-nutrients applied during the three-years experiments, which mainly depended on the composition of the fertilizer. Indeed, compared to control trees (no foliar sprayings), the foliar application of B increased its concentration in the leaves 4.5-fold. In general, leaf B content ranged from 19.5 mg Kg^−1^ DW in the control during the first year to 171 mg kg^−1^ DW in the B treated plants during the third year of the trial (Table 2), indicating that all of the plants did not show any deficiency in leaf boron concentration. Indeed, the optimum boron concentration in hazelnut leaves was indicated as ranging between 14 and 30 mg kg^−1^, depending on the cultivars and the soil composition [6,20]. 

Under CD foliar fertilization, all of the micronutrients determined in the leaves were increased compared to both control and the B treated trees (except for the Mg content). A positive effect of CD treatments was also found for the leaf P content, which displayed significantly higher levels in CD leaves, particularly during the first year of the trial. 

Foliar treatments did not affect the yield components in 2011 and 2012, while only a 21% decrease in fruits number on the B treated plants in 2013 was recorded compared to control. These results are in contrast to some research performed in Oregon [15,16,22], but in agreement with the works carried out in the Mediterranean conditions [13,18,19,25]. This discrepancy can be explained by considering both the different climatic conditions present and the different cultivars grown in these different geographical areas. Furthermore, in a recent experiment conducted on Tonda di Giffoni plants in Serbia, researchers highlighted how the combined application of boron and zinc can improve nut quality and yield [32] compared to non-sprayed control plants.

Foliar spray significantly improved kernel dry weight in CD compared to the untreated control; this result is in agreement with other trials of foliar fertilization performed in hazelnut [13,22,23,25]. This effect supports the hypothesis that foliar nutrition influences the development of shells and embryos. In particular, boron is important in the development of cell wall and cell membranes function and metabolic activities [33]. Moreover, boron and zinc are involved in the biosynthesis of auxin, which produces more plant cells and more dry matter [34]. Foliar fertilization (CD and B) decreased the percentage of blank compared to the control. This result is in full agreement with those obtained in other trials of B foliar fertilization with or without zinc [13,22,23,35]. The benefit of foliar B on blank reduction can be partially explained by an increase in pollen tube growth. Furthermore, boron is needed for the formation of tissue; therefore, foliar application prior to or immediately following bloom increases cell division and improves the fruit set [36].

### 3.2. Effects of Foliar Fertilization on Kernel Composition and Quality

The increase in elemental constituents of seed may be due to the effect of micronutrients in stimulating biological activities, such as enzyme activity, chlorophyll synthesis, the rate of translocation of photosynthetic products and the increase nutrient uptake through roots after foliar fertilization [34]. Boron is a fundamental element involved in a large number of metabolic pathways (sugar transport, respiration, carbohydrate, RNA, IAA and phenol metabolism) [37]. Boron and zinc were capable of increasing the oil percentage of olive fruits [38,39]. The increase in fat content in kernels treated with CD and B is in accordance with other several oilseed species, including cotton [40], rapeseed [41], peanut [42] and pecan [43]. These results could be attributed to the impact of boron with or without zinc on both functional and structural mechanisms of enzyme activities within plant cell compartments [37]. The higher protein content in the CD kernels is probably due to the increased availability of nitrogen. In pecan, foliar application of urea increased total nitrogen in plant tissue, which led to higher protein content in seeds due to the fact that nitrogen is a constituent of protein [44]. Furthermore, the application of zinc also increases seed protein content: Shchitaeva [45] found that the synthesis of metabolically active amino acids depends on zinc application, which increased the synthesis of asparagines and tryptophan. Similar results were obtained in pecan [43] and peanut [46]. The greater carbohydrate content in control kernels can be explained by the fact that the synthesis of lipids and proteins in the seeds occurs at the expense of carbohydrates. Analogue results were found in almond [47] and in rapeseed [48]. 

In the hazelnut kernel, fat is one of the main components, and the Mortarella cultivar has between 54% and 64.6% fat content [49]. Fat plant metabolism is quite a complex process, which mainly takes place in plastid and endoplasmic reticulum. It is hard to find similar work dealing with the effect of microelements on the fatty acid composition of hazelnuts. In this trial, boron improved the oil quality of kernels; however, it seems that fatty acid compositions responded differently to nutrients spray, as oleic acid was promoted, while palmitic acid was prohibited, under treated plants compared to control. These results were similar to those reported by Desouky et al. (2009) [39], who showed that foliar application with boron on olive might manipulate fatty acid compositions differently. Analogue response was found in olive treated with boron and zinc [38]. The higher linoleic acid content in fatty composition of CD kernels is in full agreement with Dag et al. (2009) [50] and could be explained by the nitrogen fertilization. This fatty acid modification may be caused by the enhancement or inhibition of oleate desaturase activity during triacylglycerol biosynthesis [50]. This observation could be explained by the fact that the nitrogen supply can materially assist in retaining leaves in active photosynthesis and accelerate the seed maturity; as the highest polyunsaturated fatty acid accumulation is expected in fully ripened seed, this might be a reason why nitrogen increases the content of linoleic acid [51]. This comment also explains the higher PUFA content in the CD kernels. Instead, the higher content of UFA and the lowest content of SFA of the treated plants compared to the control can be explained by the higher oleic percentage and the lowest palmitic percentage of fatty acids in foliar fertilized kernels.

Fat oxidation in hazelnuts constantly increases during product storage; therefore, it is crucial to have low levels of fat alteration at harvest time. In our work, the fat oxidation levels of fruits at harvest were quite low and in line with those found in a previous work on hazelnuts harvested over several years in the Campania region of Italy [49]. The higher lipid stability of foliar sprayed hazelnuts compared to control is mainly due to the higher content of total polyphenols. Polyphenols are important antioxidants which protect biological systems against oxygen radicals [52]; therefore, these substances can contribute certain signals for stabilizing oil content [53]. In hazelnut, the total phenol content and antioxidant status of several cultivars was already investigated by Pelvan et al. (2012) [54]. The higher total polyphenols content in foliar fertilized kernels could be explained by the role of boron in the metabolism of phenolic compounds [39]. Boron is one of nutrients responsible for the change in the concentration and metabolism of phenolic compounds in vascular plants [55]. Furthermore, boron deprivation increased the polyphenol oxidase activity, i.e., the enzyme that catalyzes the oxidation of phenolic compounds [56]. This response is in agreement with that found in olive after foliar fertilization with boron and zinc [38].

## 4. Materials and Methods

### 4.1. Plant Material and Experimental Site

The trial was conducted in a 10-year-old private hazelnut orchard (*Corylus avellana* L.), cv. ‘Mortarella’, which is grown at open vase and located in Caianello, Caserta, Southern Italy (41°18′00″ N 14°05′00″ E), during the years 2011–2013. Trees were spaced 3 m × 4 m, and tree rows were North–South oriented.

Soil samples were collected, from 0 to 30 cm depths, at the beginning of the experimental trial (late winter 2011) and analyzed for physical and chemical properties (Table 6). Based on USDA classification [57], orchard soil texture was clay-loam.

Soil fertility management (417 kg ha^−1^ 20:20:20 NPK at bud swell; 417 kg ha^−1^ urea 46% in mid-June) and other agricultural practices followed local ordinary practices. The orchard was rainfed.

### 4.2. Foliar Nutrition Treatments and Foliar Mineral Analysis

Three experimental plots of 300 m^2^ each were subjected to foliar nutrition treatments as follows: control (distilled water), Coryl-Dry Veg (8% CH_4_N_2_O, 1% N organic, 0.5% B soluble, 0.5% Zn EDTA, 0.05% Fe EDTA, Biochemie International, Giffoni Valle Piana, Italy) and Boromin 135 gel (boron ethanolamine 10%, Biolchim, Bologna, Italy). The experimental design included 3 randomized blocks of 5 trees per treatment, with a total of 15 plants treated per treatment, that were sprayed with a distilled water solution of Coryl-Dry (CD) at 2.5%, with a solution 300 ppm of boron ethanolamine (B) or with only water (Control). To avoid any derivatives, measurements were taken from the three central trees.

The spraying was performed three times per year in 2011, 2012 and 2013 with a backpack pump pressure sprayer, starting from the fifth leaf stage (14 April 2011, 17 April 2012 and 16 April 2013) and occurring every 3–4 weeks (12 May and 17 June 2011; 19 May and 13 June 2012; 12 May and 18 June 2013).

### 4.3. Foliar Analyses

Leaf micronutrient content analyses were performed once per year (June 2011, June 2012 and June 2013) by collecting three samples of five adult leaves randomly selected from the trees included in the blocks of the three treatments.

Macro and micro elements (N-NO_3_, N-NH_4_, P, K, Ca, Mg, Fe, Zn, B, and Mn) in leaf samples were measured using an inductively coupled plasma mass spectrometer (ICP-OES Spectroblue, Spectro Ametek, Berwyn, PA, USA), as reported by Pannico et al. (2019) [58]. Briefly, 1000 mg of lyophilized leaves were fully digested in a microwave digestion system (MLS-1200 Microwave Laboratory Systems, Milestone, Shelton, CT, USA) with the addition of a mixture of HNO3 (65%) and HCl (37%) (9:3, *v*/*v*; 12 mL), and the resulting solutions were transferred to 100-mL volumetric flasks and diluted to the fixed volume (50 mL) with ultrapure water (Milli-Q, Merck Millipore, Darmstadt, Germany). The calibration curve was prepared using a working standard solution, with concentrations ranging from 1.0 to 100 µg L^−1^ for all elements. The results were expressed as mg kg^−1^ dw and g 100 g^−1^ dw for micro and macro elements, respectively.

### 4.4. Yield, Nut and Kernel Biometrical Traits

At harvest (22 September 2011, 25 September 2012 and 18 September 2013), yield per tree was weighed when commercial moisture was reached (6%), and samples of 1 kg of nuts per tree were collected and stored at −20 °C until analysis, in the three experimental years.

The number of nuts per kilogram per plant was counted to estimate the number of fruits per tree. Nut and kernel dry weight were measured after being dried in a ventilated oven at 60 °C until reaching constant weights.

### 4.5. Kernel Constituents and Fatty Acid Composition

Ash, total fat, total protein, carbohydrate and total phenols of kernels subjected to different treatments were determined. Total ash was determined by incinerating the dry samples (500 mg of finely chopped kernels) for 3 h at 550 °C in muffle furnace according to the AOAC method (1995) [59]. Total fat was extracted using a Soxhlet extractor; 5 g of finely crushed kernels were placed in a cellulose thimble and extracted with 30 mL of petroleum ether (boiling point 40–60 °C) for 6 h [59]. Oil extracted was stored at −20 °C until analysis. Total crude protein was determined via the macro Kjeldahl method; protein content was calculated as Total N × 4.38. Carbohydrate content was obtained using the following formula: carbohydrate content = 100% − (% moisture + % protein + % fat + % ash), according to Olivera et al. (2008) [60]. Total phenols were determined according to the method of Jakopic et al. (2011) [61] with some modifications. Hazelnut flour (5 g) was extracted for 45 min with 30 mL of methanol/water (80:20 *v*/*v*) in a water bath using sonification. The hazelnut extracts were centrifuged at 7000 rpm for 10 min, and the supernatant was filtered through a 0.45 μm membrane filter. In total, 5 ml of extract was mixed with 5 mL n-hexane for 3 min in a vortex apparatus, and the mixture was centrifuged at 7000 rpm for 5 min to remove lipid fraction. The procedure was repeated twice with 5 mL of n-hexane. The total phenolic content of the extracts was assessed using the Folin–Ciocalteau phenol reagent method (Singleton and Rossi, 1965 [62]. Next, 10 ml of bi-distilled water and 500 μL of Folin–Ciocalteau reagent were added to 2 mL of diluted extract (1:5 in water), and 1 mL of sodium carbonate (20%, *w*/*v*) was added after 3 min. The absorbance at 765 nm was measured after 30 min in the dark. The total phenolic content was expressed as gallic acid equivalents (GAE) in mg 100 g^−1^ of hazelnut.

Fatty acid composition was analyzed via gas chromatography after derivatization to fatty acid methyl ester (FAME), according to the IUPAC standard method [63] slightly modified following Pannico et al. [64]. A GC Perkin Elmer AutoSystem XL (PerkinElmer, MA, USA.) equipped with a programmed temperature vaporizer, a flame ionization detector (FID), and a capillary column with 100 m × 0.25 mm ID and a film thickness of 0.20 μm using a stationary phase of 50% cianopropyl methyl silicone (Supelco, Bellofonte, PA, USA) was used. The carrier gas, i.e., helium, was introduced at a flow rate of 20 cm/s. The oven temperature program was as follows: 120 °C for 5 min, 5 °C/min ramp-up to 165 °C for 5 min, and then 10 °C/min ramp-up to 240 °C for 20 min. The split ratio was 1/60, and the FID temperature was 260 °C. Fatty acids were identified via comparison with retention times of external standards (SupelcoTM 37 component FAME MIX). Fatty acid concentrations were calculated through a comparison with the pure standard retention time and were based on response factors used to convert peak areas into weight percentages.

### 4.6. Analyses of Lipid Alteration and Oxidation

The detection of primary and secondary oxidation was performed spectrophotometrically [65]. This analysis considered the measurements of two variables: K_232_ and K_270_. K_232_ was a measure of the level of conjugated dienes and was indicative of the primary oxidation. K_270_ was a measure of the level of conjugated trienes, which was indicative of the secondary oxidation. UV specific extinction determination permitted a good approximation of the oxidation process in unsaturated oils [65,66]. The specific extinction coefficients, which were set at 232 nm and 270 nm, were measured according to the following procedure. An oil sample of 100 mg was placed in a 10 mL flask and diluted to 10 mL with spectrophotometric grade hexane (Sigma-Aldrich). The sample was then homogenized, and the absorbance was measured with a UV-Vis 4000 spectrophotometer (Varian, Palo Alto, CA, USA) using pure solvent as blank [67]. Free fatty acids (FFA) were measured via direct titration of the nuts oil extract with 0.1 N NaOH, using phenolphthalein as an indicator. Free fatty acid contents of oil samples were determined in accordance with method no. 2.201 of IUPAC (1987) [63]. The peroxide value (PV) was determined using the extracted oil and estimated via iodometric titration assay, which is based on the oxidation of the iodide ion using hydroperoxides (ROOH). A saturated solution of potassium iodide was added to oil samples to react with hydroperoxides. The liberated iodine was then titrated with a standardized solution of sodium thiosulfate and starch as an endpoint indicator. The PV was obtained via calculation and reported as milliequivalents of oxygen per kilogram of samples (meq/kg); the official determination was described in method no. 2.501 of IUPAC (1987) [63]. All analyzes were performed in triplicate.

### 4.7. Statistical Analysis

All data were subjected to bifactorial analysis of variance (two-way ANOVA) (year (Y) ×fertilization (F)), using a general linear model generated using the SPSS software package (SPSS version 22, Chicago, IL, USA). Mean effects and interactions were separated according to Tukey’s HSD test (*p* = 0.05). For Table 3, Table 4 and Table 5, the mean effects of the two years were compared according to Student’s *t*-test.

## 5. Conclusions

As demonstrated in some previous experiences, in hazelnut, foliar nutrition improves some characteristics of plant production and kernel quality, which, in turn, can positively affect the commercial value of yield. Integrated foliar application of nitrogen and micronutrients, such as boron and zinc, may enhance some qualitative and nutritional characteristics of nuts at harvest, namely kernel dry weight, fat content, and fatty acid composition, with the latter enhanced by increasing the relative content of monounsaturated fatty acids. However, despite the higher concentration of MUFA, foliar nutrient applications, mainly CD, positively influenced kernel fat stability during oxidation, with a relevant effect on hazelnut storability also recorded. Kernel produced under foliar nutrition sprayings were probably resulted more protected from fatty acid oxidation due to a higher concentration of polyphenols. Since the applied foliar fertilization treatments differentially affected the yield and nutritional value of hazelnut cultivar Mortarella, which is grown in the cultivation area of Southern Italy, it appears valuable to consider the possibility of integrating the ordinary practices of orchard nutrition management using complex foliar fertilizers, instead of boron foliar sprayings, to obtain a more economically valuable and healthy production of nuts, both at harvest and during the post-harvest period.

## Figures and Tables

**Table 1:** Yield components, which are expressed in kg and number of nuts per plant, percentage of blank nuts and nut and kernel weight of foliar fertilized plants (CD and B) and control plants (C) at harvest in 2011, 2012 and 2013. Data are expressed as mean ± s.e. (n = 3).

Source of Variance	Yield	Nut Number	Blank	Nut Weight	Kernel Dry Weight
	(kg Plant^−1^)	(no. Plant^−1^)	(%)	(g)	(g)
Year (Y)					
2011	3.29 ± 0.16 b	1357 ± 48.37 b	4.42 ± 0.34 b	2.42 ± 0.06 b	1.07 ± 0.01 b
2012	3.30 ± 0.10 b	1245 ± 41.66 b	3.87 ± 0.53 c	2.66 ± 0.05 a	1.21 ± 0.02 a
2013	4.36 ± 0.26 a	1613 ± 99.45 a	5.17 ± 0.34 a	2.71 ± 0.02 a	1.24 ± 0.01 a
	***	***	***	***	***
Fertilization (F)					
Control	3.56 ± 0.21 b	1400 ± 72.83 b	5.86 ± 0.19 a	2.55 ± 0.07	1.14 ± 0.03 b
CD	4.16 ± 0.29 a	1557 ± 103.1 a	4.04 ± 0.25 b	2.67 ± 0.04	1.20 ± 0.03 a
B	3.23 ± 0.13 b	1257 ± 36.32 c	3.57 ± 0.39 c	2.57 ± 0.07	1.17 ± 0.03 ab
	***	***	***	ns	*
Y × F					
2011 × Control	3.24 ± 0.05 cd	1418 ± 30.77 bcd	5.54 ± 0.18 ab	2.28 ± 0.05 c	1.04 ± 0.01
2011 × CD	3.86 ± 0.03 bc	1472 ± 43.30 bc	3.23 ± 0.08 e	2.62 ± 0.06 ab	1.10 ± 0.01
2011 × B	2.77 ± 0.12 d	1179 ± 31.91 cd	4.49 ± 0.10 cd	2.35 ± 0.04 bc	1.07 ± 0.02
2012 × Control	3.08 ± 0.06 cd	1156 ± 46.18 d	5.60 ± 0.40 ab	2.67 ± 0.06 a	1.19 ± 0.01
2012 × CD	3.45 ± 0.25 cd	1273 ± 60.58 cd	3.96 ± 0.20 de	2.70 ± 0.09 a	1.23 ± 0.04
2012 × B	3.37 ± 0.17 cd	1305 ± 94.16 cd	2.05 ± 0.09 f	2.60 ± 0.11 abc	1.21 ± 0.03
2013 × Control	4.37 ± 0.16 ab	1627 ± 69.58 ab	6.43 ± 0.07 a	2.69 ± 0.02 a	1.20 ± 0.02
2013 × CD	5.17 ± 0.37 a	1925 ± 101.1 a	4.93 ± 0.07 bc	2.68 ± 0.05 a	1.26 ± 0.03
2013 × B	3.56 ± 0.08 bcd	1286 ± 36.80 cd	4.17 ± 0.16 cd	2.77 ± 0.05 a	1.24 ± 0.01
	*	***	***	*	ns

ns, *, **, *** non-significant or significant at *p* ≤ 0.05, 0.01, and 0.001, respectively. Different letters within each column indicate significant differences according to Tukey’s HSD test (*p* = 0.05).

**Table 2:** Leaf mineral composition of foliar fertilized plants (CD and B) and control plants (C) in summer 2011, 2012 and 2013. Data are expressed as mean ± s.e., (n = 3).

Source of Variance	NO_3_	NH_4_	P	K	Ca	Mg	Zn	Fe	B	Mn
	(g 100 g^−1^ DW)	(g 100 g^−1^ DW)	(g 100 g^−1^ DW)	(g 100 g^−1^ DW)	(g 100 g^−1^ DW)	(g 100 g^−1^ DW)	(mg kg^−1^ DW)	(mg kg^−1^ DW)	(mg kg^−1^ DW)	(mg kg^−1^ DW)
Year (Y)										
2011	0.023 ± 0.002	0.024 ± 0.001 c	0.233 ± 0.02 a	0.815 ± 0.01 ab	0.752 ± 0.02 a	0.188 ± 0.01 a	23.21 ± 1.99 b	135.5 ± 7.02 a	37.87 ± 5.25 c	569.4 ± 27.1 a
2012	0.015 ± 0.002	0.038 ± 0.002 a	0.225 ± 0.01 a	0.888 ± 0.03 a	0.607 ± 0.03 b	0.161 ± 0.01 b	34.17 ± 4.83 a	119.9 ± 5.15 b	49.04 ± 9.80 b	453.5 ± 18.0 b
2013	0.017 ± 0.002	0.029 ± 0.001 b	0.147 ± 0.01 b	0.776 ± 0.03 b	0.534 ± 0.02 b	0.131 ± 0.01 c	24.89 ± 2.95 b	55.59 ± 7.10 c	79.79 ± 23.11 a	359.8 ± 5.39 c
	ns	***	***	*	***	***	***	***	***	***
Fertilization (F)										
Control	0.018 ± 0.003	0.032 ± 0.003	0.181 ± 0.02 b	0.828 ± 0.02	0.596 ± 0.04	0.148 ± 0.01 b	18.17 ± 0.74 b	86.93 ± 13.1 c	22.79 ± 1.24 c	432.9 ± 15.5 c
CD	0.018 ± 0.003	0.029 ± 0.002	0.218 ± 0.02 a	0.789 ± 0.03	0.648 ± 0.04	0.172 ± 0.01 a	31.68 ± 2.38 a	118.1 ± 9.64 a	39.19 ± 2.11 b	489.7 ± 39.4 a
B	0.020 ± 0.001	0.029 ± 0.002	0.206 ± 0.02 ab	0.862 ± 0.03	0.649 ± 0.04	0.160 ± 0.01 ab	32.43 ± 4.73 a	105.9 ± 15.8 b	104.7 ± 17.32 a	460.2 ± 43.0 b
	ns	ns	*	ns	ns	*	***	***	***	***
Y × F										
2011 × control	0.021 ± 0.003	0.025 ± 0.002	0.180 ± 0.02 bcd	0.786 ± 0.02	0.745 ± 0.01	0.180 ± 0.01	16.92 ± 0.86 e	111.9 ± 3.85 c	19.46 ± 0.58 g	463.9 ± 17.6 c
2011 × CD	0.026 ± 0.006	0.025 ± 0.001	0.281 ± 0.02 a	0.799 ± 0.01	0.765 ± 0.04	0.203 ± 0.01	22.48 ± 1.26 d	136.1 ± 4.00 b	38.50 ± 0.31 de	613.6 ± 0.40 a
2011 × B	0.022 ± 0.002	0.021 ± 0.001	0.239 ± 0.03 ab	0.860 ± 0.02	0.745 ± 0.05	0.181 ± 0.01	30.24 ± 0.70 c	158.5 ± 4.09 a	55.64 ± 1.66 c	630.7 ± 8.86 a
2012 × control	0.012 ± 0.005	0.043 ± 0.001	0.226 ± 0.02 abc	0.864 ± 0.03	0.539 ± 0.04	0.143 ± 0.01	16.70 ± 0.34 e	114.1 ± 1.14 bc	27.40 ± 0.83 efg	459.4 ± 4.70 c
2012 × CD	0.014 ± 0.006	0.034 ± 0.003	0.220 ± 0.02 abcd	0.865 ± 0.06	0.624 ± 0.04	0.176 ± 0.01	36.14 ± 1.16 b	136.1 ± 10.7 b	32.29 ± 0.49 ef	512.1 ± 4.48 b
2012 × B	0.020 ± 0.001	0.036 ± 0.002	0.229 ± 0.01 abc	0.934 ± 0.06	0.660 ± 0.05	0.165 ± 0.01	49.67 ± 1.87 a	109.5 ± 0.38 c	87.43 ± 6.39 b	389.1 ± 7.56 d
2013 × control	0.020 ± 0.004	0.029 ± 0.001	0.138 ± 0.01 d	0.834 ± 0.03	0.505 ± 0.04	0.120 ± 0.01	20.88 ± 0.42 de	34.80 ± 1.80 e	21.52 ± 0.66 fg	375.3 ± 6.48 de
2013 × CD	0.013 ± 0.001	0.027 ± 0.002	0.154 ± 0.02 bcd	0.704 ± 0.06	0.555 ± 0.03	0.137 ± 0.01	36.40 ± 1.21 b	82.16 ± 3.76 d	46.79 ± 0.02 cd	343.4 ± 2.23 e
2013 × B	0.019 ± 0.003	0.030 ± 0.001	0.150 ± 0.00 cd	0.791 ± 0.05	0.541 ± 0.02	0.135 ± 0.01	17.39 ± 0.57 de	49.82 ± 1.30 e	171.1 ± 0.74 a	360.9 ± 6.85 de
	ns	ns	*	ns	ns	ns	***	***	***	***

ns, *, **, *** non-significant or significant at *p* ≤ 0.05, 0.01, and 0.001, respectively. Different letters within each column indicate significant differences according to Tukey’s HSD test (*p* = 0.05).

**Table 3:** Composition of kernel dry matter (fat, carbohydrate, protein and ash content, expressed as grams per 100 g of kernel dry weight) for hazelnut harvested in 2012 and 2013 from foliar fertilized plants (CD and B) and control plants (C). Data are expressed as mean ± s.e., (n = 3).

Source of Variance	Fat Content	Protein Content	Carbohydrate Content	Ash Content
	(g 100 g^−1^ DW)	(g 100 g^−1^ DW)	(g 100 g^−1^ DW)	(g 100 g^−1^ DW)
Year (Y)				
2012	63.47 ± 0.77	16.45 ± 0.81	17.81 ± 1.34	2.28 ± 0.03
2013	62.20 ± 0.63	16.47 ± 0.77	19.24 ± 1.19	2.08 ± 0.06
*t*-test	ns	ns	ns	**
Fertilization (F)				
Control	60.18 ± 0.26 b	14.77 ± 0.43 b	22.94 ± 0.60 a	2.10 ± 0.08
CD	63.80 ± 0.39 a	19.47 ± 0.28 a	14.59 ± 0.55 c	2.15 ± 0.06
B	64.53 ± 0.48 a	15.14 ± 0.14 b	18.04 ± 0.56 b	2.29 ± 0.04
	***	***	***	ns
Y × F				
2012 × control	60.54 ± 0.33	14.71 ± 0.94	22.51 ± 1.24	2.24 ± 0.03
2012 × CD	64.41 ± 0.30	19.45 ± 0.42	13.87 ± 0.66	2.27 ± 0.04
2012 × B	65.44 ± 0.54	15.19 ± 0.19	17.05 ± 0.76	2.32 ± 0.06
2013 × control	59.81 ± 0.29	14.84 ± 0.11	23.38 ± 0.25	1.97 ± 0.10
2013 × CD	63.18 ± 0.53	19.49 ± 0.47	15.30 ± 0.76	2.03 ± 0.06
2013 × B	63.62 ± 0.20	15.10 ± 0.24	19.03 ± 0.06	2.25 ± 0.05
	ns	ns	ns	ns

ns, *, **, *** non-significant or significant at *p* ≤ 0.05, 0.01, and 0.001, respectively. Different letters within each column indicate significant differences according to Tukey’s HSD test (*p* = 0.05). Two years were compared according to Student’s *t*-test.

**Table 4:** Quality and stability of oil extracted from kernels of hazelnut harvested in 2012 and 2013 from foliar fertilized plants (CD and B) and control plants (control): free acidity, peroxide value and K_232_ and K_270_ absorption coefficients are shown. Data are expressed as mean ± s.e., (n = 3).

Source of Variance	Free Acidity	Peroxide Value	K_232_	K_270_
	(% Oleic Acid)			
Year (Y)				
2012	1.05 ± 0.01	2.31 ± 0.56	1.61 ± 0.04	0.36 ± 0.05
2013	0.42 ± 0.06	7.04 ± 0.21	1.83 ± 0.10	0.87 ± 0.08
*t*-test	***	***	ns	***
Fertilization (F)				
Control	0.87 ± 0.09 a	5.99 ± 0.73 a	1.93 ± 0.09 a	0.83 ± 0.12 a
CD	0.66 ± 0.17 b	3.55 ± 1.36 c	1.48 ± 0.02 c	0.40 ± 0.08 c
B	0.69 ± 0.16 b	4.48 ± 1.11 b	1.74 ± 0.08 b	0.61 ± 0.15 b
	***	***	***	***
Y × F				
2012 × control	1.07 ± 0.01 a	4.37 ± 0.03 c	1.76 ± 0.05 bc	0.56 ± 0.01 c
2012 × CD	1.04 ± 0.01 a	0.53 ± 0.03 e	1.50 ± 0.01 d	0.22 ± 0.02 d
2012 × B	1.05 ± 0.01 a	2.03 ± 0.02 d	1.57 ± 0.01 cd	0.29 ± 0.01 d
2013 × control	0.66 ± 0.01 b	7.62 ± 0.07 a	2.11 ± 0.06 a	1.10 ± 0.01 a
2013 × CD	0.28 ± 0.01 d	6.57 ± 0.31 b	1.47 ± 0.03 d	0.58 ± 0.03 c
2013 × B	0.33 ± 0.02 c	6.93 ± 0.38 ab	1.91 ± 0.05 b	0.94 ± 0.03 b
	***	***	***	***

ns, *, **, *** non-significant or significant at *p* ≤ 0.05, 0.01, and 0.001, respectively. Different letters within each column indicate significant differences according to Tukey’s HSD test (*p* = 0.05). Two years were compared according to Student’s *t*-test.

**Table 5:** Relative content of fatty acids in oil extracted from kernels of hazelnuts harvested in 2012 and 2013 from foliar fertilized plants (CD and B) and control plants (control). All data are expressed as mean ± s.e., (n = 3).

Fatty Acids (%)	Year (Y)			Fertilization (F)				Y × F						
	2012	2013	*t*-Test	Control	CD	B	Sig.	2012 × Control	2012 × CD	2012 × B	2013 × Control	2013 × CD	2013 × B	Sig.
C16:0	5.98 ± 0.02	5.85 ± 0.09	ns	6.10 ± 0.04 a	5.80 ± 0.09 b	5.83 ± 0.04 b	***	6.03 ± 0.01 b	6.00 ± 0.05 b	5.92 ± 0.04 b	6.18 ± 0.03 a	5.61 ± 0.00 d	5.75 ± 0.01 c	***
C16:1	0.18 ± 0.004	0.18 ± 0.008	ns	0.19 ± 0.008 a	0.17 ± 0.004 b	0.17 ± 0.007 b	**	0.18 ± 0.011 abc	0.18 ± 0.007 abc	0.18 ± 0.006 ab	0.20 ± 0.002 a	0.17 ± 0.002 bc	0.15 ± 0.002 c	***
C17:0	0.043 ± 0.001	0.037 ± 0.001	**	0.042 ± 0.002	0.038 ± 0.002	0.039 ± 0.002	ns	0.043 ± 0.004	0.043 ± 0.001	0.042 ± 0.001	0.041 ± 0.000	0.033 ± 0.001	0.035 ± 0.002	ns
C17:1	0.066 ± 0.001	0.057 ± 0.001	***	0.063 ± 0.002	0.060 ± 0.003	0.060 ± 0.002	ns	0.067 ± 0.002	0.065 ± 0.003	0.065 ± 0.001	0.060 ± 0.000	0.055 ± 0.002	0.055 ± 0.001	ns
C18:0	3.60 ± 0.05	2.65 ± 0.04	***	3.21 ± 0.24 a	3.08 ± 0.25 b	3.10 ± 0.15 ab	*	3.74 ± 0.07 a	3.64 ± 0.01 a	3.43 ± 0.05 b	2.67 ± 0.05 cd	2.52 ± 0.01 d	2.77 ± 0.02 c	***
C18:1 n9 c	82.56 ± 0.06	83.48 ± 0.08	***	82.80 ± 0.17 c	83.03 ± 0.25 b	83.23 ± 0.20 a	***	82.44 ± 0.07 d	82.46 ± 0.04 d	82.79 ± 0.05 c	83.17 ± 0.05 b	83.60 ± 0.02 a	83.67 ± 0.00 a	**
C18:1 n9 t	0.019 ± 0.002	0.015 ± 0.001	ns	0.017 ± 0.003	0.017 ± 0.001	0.017 ± 0.001	ns	0.021 ± 0.005	0.017 ± 0.003	0.018 ± 0.000	0.013 ± 0.000	0.017 ± 0.000	0.016 ± 0.002	ns
C18:2 n6 c	7.08 ± 0.02	7.31 ± 0.07	**	7.12 ± 0.05 b	7.35 ± 0.10 a	7.11 ± 0.01 b	***	7.02 ± 0.01 d	7.13 ± 0.02 c	7.10 ± 0.01 cd	7.22 ± 0.03 b	7.58 ± 0.00 a	7.13 ± 0.02 c	***
C18:3 n3	0.11 ± 0.001	0.12 ± 0.003	*	0.12 ± 0.004 b	0.12 ± 0.003 a	0.11 ± 0.002 c	***	0.11 ± 0.001 c	0.12 ± 0.002 b	0.12 ± 0.002 b	0.13 ± 0.001 a	0.13 ± 0.001 a	0.11 ± 0.001 c	***
C18:3 n6	0.15 ± 0.002	0.11 ± 0.002	***	0.13 ± 0.007 a	0.13 ± 0.012 ab	0.12 ± 0.008 b	*	0.15 ± 0.003 a	0.15 ± 0.004 a	0.14 ± 0.001 a	0.12 ± 0.000 b	0.10 ± 0.003 c	0.11 ± 0.003 bc	**
C20:1	0.12 ± 0.003	0.14 ± 0.004	***	0.14 ± 0.003	0.13 ± 0.005	0.13 ± 0.010	ns	0.13 ± 0.005 bcd	0.12 ± 0.004 cd	0.11 ± 0.001 d	0.14 ± 0.000 ab	0.14 ± 0.003 bc	0.16 ± 0.007 a	**
C20:3 n6	0.030 ± 0.001	0.026 ± 0.001	***	0.030 ± 0.001 b	0.027 ± 0.002 b	0.028 ± 0.001 a	***	0.032 ± 0.001 a	0.031 ± 0.001 ab	0.029 ± 0.000 abc	0.028 ± 0.000 bc	0.023 ± 0.001 d	0.026 ± 0.000 c	***
C23:0	0.049 ± 0.002	0.027 ± 0.000	***	0.037 ± 0.004	0.040 ± 0.006	0.038 ± 0.005	ns	0.045 ± 0.002 a	0.052 ± 0.003 a	0.050 ± 0.001 a	0.028 ± 0.001 b	0.027 ± 0.000 b	0.026 ± 0.001 b	*
SFA	9.68 ± 0.07	8.56 ± 0.11	***	9.39 ± 0.21 a	8.96 ± 0.34 b	9.01 ± 0.19 b	***	9.86 ± 0.07 a	9.73 ± 0.05 a	9.44 ± 0.05 b	8.92 ± 0.07 c	8.19 ± 0.01 e	8.58 ± 0.03 d	***
UFA	90.32 ± 0.07	91.44 ± 0.11	***	90.61 ± 0.21 b	91.04 ± 0.34 a	90.99 ± 0.19 a	***	90.14 ± 0.07 e	90.27 ± 0.05 e	90.56 ± 0.05 d	91.08 ± 0.07 c	91.81 ± 0.01 a	91.42 ± 0.03 b	***
MUFA	82.95 ± 0.06	83.87 ± 0.07	***	83.21 ± 0.17 c	83.41 ± 0.25 b	83.61 ± 0.20 a	***	82.83 ± 0.08 d	82.84 ± 0.04 d	83.17 ± 0.05 c	83.59 ± 0.05 b	83.97 ± 0.02 a	84.05 ± 0.01 a	**
PUFA	7.37 ± 0.02	7.57 ± 0.07	*	7.40 ± 0.04 b	7.63 ± 0.09 a	7.38 ± 0.01 b	***	7.31 ± 0.01 d	7.43 ± 0.02 bc	7.39 ± 0.01 cd	7.49 ± 0.03 b	7.83 ± 0.01 a	7.37 ± 0.02 cd	***
Omega 3	0.11 ± 0.001	0.12 ± 0.003	*	0.12 ± 0.004 b	0.12 ± 0.003 a	0.11 ± 0.002 c	***	0.11 ± 0.001 c	0.12 ± 0.002 b	0.12 ± 0.002 b	0.13 ± 0.001 a	0.13 ± 0.001 a	0.11 ± 0.001 c	***
Omega 6	7.26 ± 0.02	7.44 ± 0.07	*	7.28 ± 0.04 b	7.51 ± 0.09 a	7.26 ± 0.01 b	***	7.20 ± 0.01 d	7.31 ± 0.02 bc	7.27 ± 0.01 cd	7.36 ± 0.03 b	7.70 ± 0.01 a	7.26 ± 0.02 cd	***

ns, *, **, *** non-significant or significant at *p* ≤ 0.05, 0.01, and 0.001, respectively. Different letters within each row indicate significant differences according to Tukey’s HSD test (*p* = 0.05). Two years were compared according to Student’s *t*-test.

**Table 6:** Physical and chemical properties of soil sampled from private hazelnut orchard located in Caianello, Caserta, Southern Italy.

Description	Units	Value
Sand, coarse	%	24
Sand, fine	%	11
Silt	%	34
Clay	%	31
pH	-	5.23
Electric conductivity	mS/cm	0.104
Organic carbon	%	1.36
Organic matter	%	2.34
Total nitrogen	%	0.15
NO_3-_	ppm	20.00
NH_3_	ppm	21.00
P_2_O_5_	ppm	61.83
K_2_O	ppm	490.20
Boron	ppm	1.1
Iron	ppm	32
Manganese	ppm	0.7
Copper	ppm	2.4
Zinc	ppm	0.5

## Data Availability

The data presented in this study are available on request from the corresponding author.
